# Hemorrhagic Pericardial Effusion as the Presenting Symptom of Newly Diagnosed Rheumatoid Arthritis

**DOI:** 10.7759/cureus.31123

**Published:** 2022-11-05

**Authors:** Eric J Basile, Iain Thompson, Omar Rafa, Megan E Hanna, Nikita J Sareen

**Affiliations:** 1 Internal Medicine, University of Florida College of Medicine, Gainesville, USA; 2 Internal Medicine, University at Buffalo, Buffalo, USA

**Keywords:** cardiology, cardiac echo, cardiac manifestation of rheumatoid arthritis, hemorrhagic pericardial effusion, complication of rheumatoid arthritis

## Abstract

Hemorrhagic pericardial effusion is a rare presenting sign of undiagnosed rheumatoid arthritis (RA). We present a case of a 58-year-old female with a history of mucinous cystadenoma with subsequent omental caking status-post small bowel resection, chronic intermittent bilateral knee pain, carpal tunnel syndrome of the left hand, and drainage of a peritoneal inclusion cyst two days prior to admission. The patient had pleuritic chest pain and acute-onset shortness of breath but was hemodynamically stable on presentation. Transthoracic echocardiogram and CT scan demonstrated a large pericardial effusion measuring 1.5 cm anteriorly, 2.21 cm posteriorly, and 2.5 cm laterally. Diagnostic pericardiocentesis revealed a hemorrhagic pericardial fluid with a glucose level of 133 mg/dL, pH of 7.34, albumin of 2.6 g/dL, red blood cell count of 401,000 cells per cubic millimeters (CUMM), white blood cell count of 1,400 CUMM, lactate dehydrogenase (LDH) of 930 U/L, and protein of 5 g/dL. Infectious and malignancy workups were negative. Rheumatologic workup was positive for elevated rheumatoid factor and anti-cyclic citrullinated peptide. The patient was diagnosed with RA; she was started on methotrexate with folic acid, and a pericardial drain was kept in place for three days. We present a brief review of the workup, etiologies, and therapeutic approach for patients who present with hemorrhagic pericardial effusion secondary to undiagnosed RA.

## Introduction

Pericardial effusion, whether it be transudative, exudative, or sanguineous, is present in up to 6.5% of the general population today in the United States [1]. The most common etiologies of new-onset hemorrhagic pericardial effusion are malignancy, infectious causes, rheumatologic diseases, and trauma. Systemic lupus erythematosus, rheumatoid arthritis (RA), and Sjogren’s syndrome are among the most common rheumatologic and inflammatory culprits. With a lifetime risk of 3.6% in women and 1.7% in men, RA leads to progressive disability and morbidity in a large subset of patients [2]. When evaluating for RA, the diagnosis can be challenging given the varied clinical presentation as well as the lack of consensus on a pathognomonic laboratory test worldwide. In patients with confirmed RA, it is estimated that the presence of pericardial effusions can be found in as high as 30% of patients with confirmed RA [3]. These pericardial effusions are typically exudative in nature with low glucose concentrations, and complement 3 and complement 4 levels. Additionally, the fluid often demonstrates high levels of lactate dehydrogenase (LDH) and gamma globulin concentrations. RA leads to a hemorrhagic pericardial effusion only in very rare cases - and hemorrhagic pericardial effusion as a presenting symptom is even rarer.

In the recent research literature, various studies and reviews have explored the relationship between RA and pericardial effusions. One such study involved an echocardiographic analysis of 101 randomly selected patients with RA identifying the prevalence of cardiac abnormalities among RA patients. Thirty-one patients were found to have 45 echocardiographic abnormalities and five patients were found to have a pericardial effusion [4]. In another systematic review, RA demonstrated a significant association with pericardial effusion, with an odds ratio of 10.7 and a 95% confidence interval of 5.0-23.0 [5]. More specifically, when looking at distinct alterations on echocardiography in patients with RA in the absence of cardiac disease symptoms, Corrao et al. identified posterior pericardial effusion, aortic root alterations, and valvular thickening as the most common findings [6]. In clinical practice today, knowledge about the presence of unrecognized cardiac abnormalities may be crucial for an accurate assessment and management of patients with RA. We describe a case of a patient presenting with an unusual type of hemorrhagic pericardial effusion secondary to undiagnosed RA.

## Case presentation

**Figure 1:**
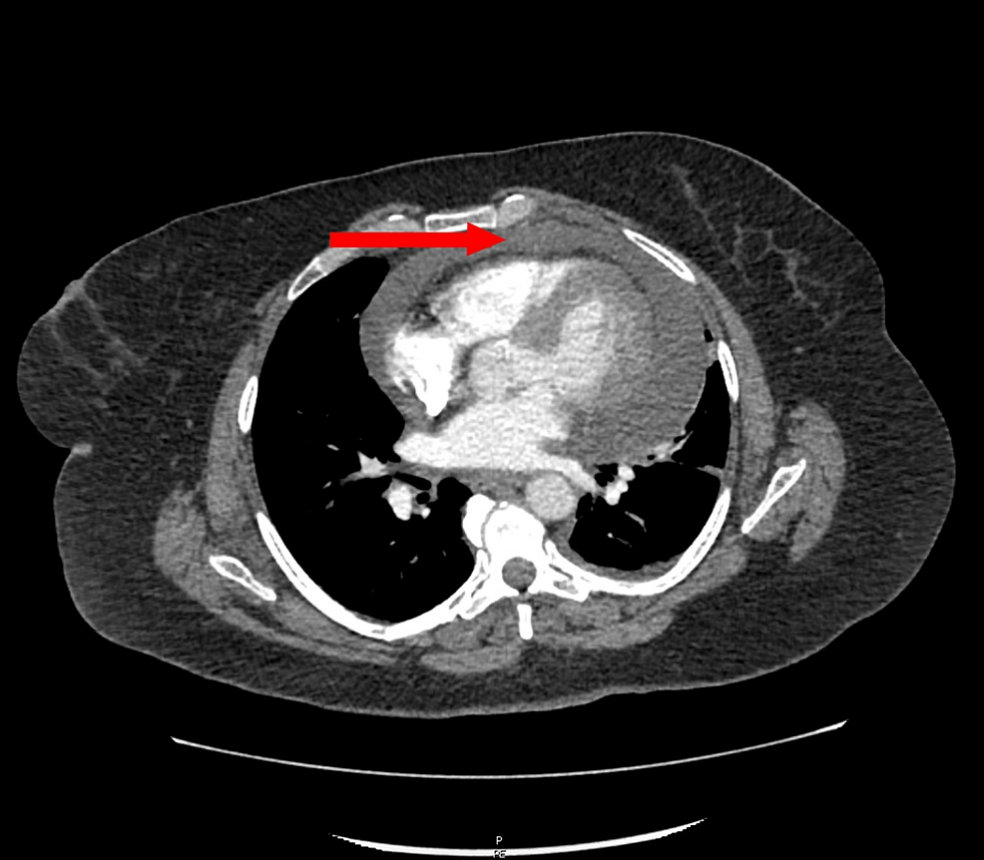
CT of the chest showing pericardial effusion (red arrow) CT: computed tomography

**Figure 2:**
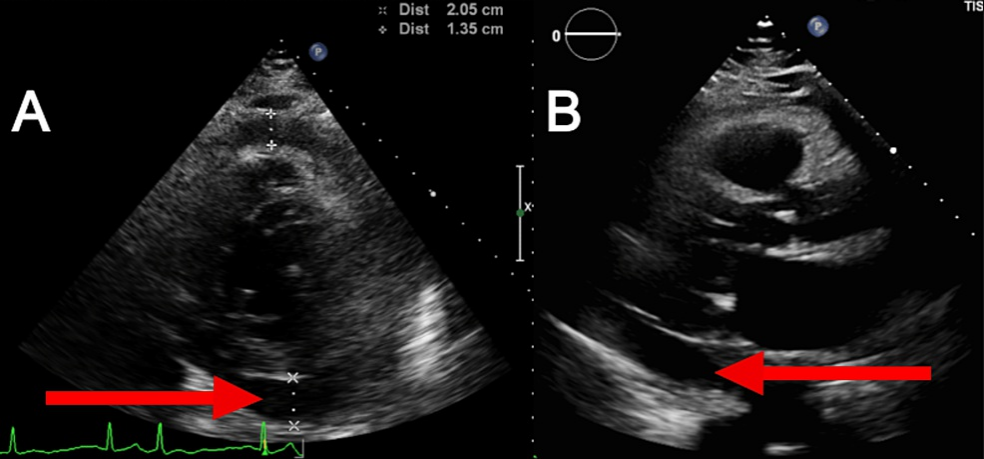
Echocardiogram of the patient A. Echocardiogram demonstrating pericardial effusion (red arrow) in the parasternal short-axis view. B. Echocardiogram demonstrating large pericardial effusion (red arrow) in the parasternal long-axis view

A 58-year-old female presented to the emergency department with complaints of chest pain and shortness of breath two days after peritoneal inclusion cyst drainage. She had pleuritic chest pain of two days’ duration with associated shortness of breath. In the emergency department, she reported moderate lower abdominal pain, occasional bilateral knee pain, chronic left wrist numbness, and left-hand pain. Her past medical history was remarkable for hypertension, chronic kidney disease stage 3A, hypothyroidism, depression, and multiple abdominal surgeries with segments of bowel removed secondary to a benign ovarian mass that led to omental caking with subsequent bowel obstruction. A review of systems was positive for fatigue, shortness of breath, chest pain, nausea, abdominal pain, chronic hematuria, occasional arthralgias, and dizziness. She recently had a low-grade fever at 100.1 ˚F for one day, was tachycardic at 110 beats per minute, had a normal respiratory rate, an oxygen saturation of 97% on room air, and a blood pressure of 136/79 mmHg. Follow-up EKGs revealed atrial flutter with predominant 2:1 AV block, ST elevation in the lateral leads, and a prolonged QTc at 519. CT of the chest revealed a large pericardial effusion (measuring 1.5 cm anteriorly, 2.21 cm posterior, and 2.5cm laterally) (Figure 1). A transthoracic echocardiogram was performed and showed normal left ventricular size, left ventricular systolic dysfunction with an ejection fraction of 40-45%, mildly dilated left atrium, mild aortic regurgitation, moderate aortic stenosis, and a large pericardial effusion (Figures 2A, 2B). Vagal maneuvers broke the arrhythmia and the patient converted to normal sinus rhythm.

The patient was admitted to the cardiac service and a diagnostic pericardiocentesis was performed, which revealed a hemorrhagic pericardial effusion. The pericardial fluid analysis demonstrated an elevated white blood cell count of 1,400 CUMM, a glucose of 133 mg/dL, protein of 5 g/dL, albumin of 2.6 g/dL, LDH of 930 U/L, pH of 7.34, and red blood cell count of 401,000 CUMM. A drain was left in situ, which drained more than 500 mL on the first day, and was subsequently removed after the third day wherein the volume threshold for removal was met. A repeat transthoracic echocardiogram was performed, which showed a significant reduction in the pericardial effusion and an improved ejection fraction of 50-55%. Bacterial cultures, gram stains, fungal cultures, and acid-fast cultures of the pericardial fluid were negative. Cytology was negative for malignancy, as were CEA, CA19-9, and CA125. Anti-CREST, anti-Scl70, anti-SSA, anti-SSB, anti-dsDNA, anti-Smith, anti-HCV, anti-Jo1, anti-Ku, anti-Mi-2, ANA, HLA-B27, complement 3, complement 4, RP, p155/140, pANCA, PL-7, PL-12, anti-Scl100, anti-SRP, anti-RNP, anti-SAE 1, anti-NXP, anti-MDA, TIF 1-gamma, and myositis panels were within normal limits. Rheumatoid factor and anti-cyclic citrullinated peptide were positive at 1,128 IU/mL and 220 U, respectively. Additionally, anti-Proteinase 3 (cANCA) was positive at 20 AU/mL, as were IgA at 469 mg/dL and IgG4 at 155 mg/dL.

A diagnosis of hemorrhagic pericardial effusion secondary to RA was made and the patient was started on disease-modifying antirheumatic drug (DMARD) therapy in the form of weekly methotrexate and folic acid. Her symptoms resolved shortly thereafter and the patient underwent sclerotherapy for the prevention of the recurrence of inclusion cysts.

## Discussion

The most common cardiac manifestation of RA is pericarditis, which can occasionally lead to exudative pericardial effusions. However, these are rarely the presenting signs of undiagnosed RA. As pericarditis and pericardial effusions are associated with increased mortality rates in RA patients, it is important to include immunologic etiologies in the differentials [7]. Even rarer is RA-associated hemorrhagic pericardial effusion with normal glucose and normal complement 3/4 levels as the presenting sign of undiagnosed RA.

Malignancy, infection, trauma, and immunologic/autoimmunity are the most common causes of hemorrhagic pericardial effusions. In cases where there is no known cause based on history and no obvious infectious etiology, RA should be included in the differentials and workup. The absence of clear joint disease should also not rule out RA, nor does the degree of joint disease appear to influence the likelihood of pericarditis and pericardial effusion [8,9]. It should also be noted that the administration of TNF-alpha inhibitors for preexisting RA does not appear to decrease the risk for pericardial effusions [8]. A thorough workup for each of the above etiologies should be performed as pericardial effusions are likely to recur and can cause a tamponade. Pericardial fluid should be collected and analyzed, especially if there is a significant collection of fluid. However, as emphasized by this case, fluid analysis is not always the most reliable method for differentiating between etiologies; for example, the traditional pericardial fluid analysis for most cases of RA-induced pericardial effusion shows minimal red blood cells, low glucose, low complement C3 and C4, elevated LDH, elevated gamma globulin, and elevated leukocytes [3]. In our case, there was a marked elevation of red blood cells and the glucose was unusually normal, as was the complement C3, C4, and gamma globulin.

RA-induced pericardial effusion can often present with tamponade as the initial accumulation of fluid is usually asymptomatic. Research literature indicates that indolent accumulation of fluid usually takes more volume in order to cause tamponade than in an acute accumulation. This makes new acute flare-ups of RA with large pericardial effusions particularly dangerous as the fluid accumulation is quicker and more likely to lead to pericardial tamponade [10]. Paradoxically, once the diagnosis is made, RA-induced pericardial effusions respond well to treatment with pericardiocentesis followed either by NSAID or colchicine as required.

## Conclusions

Early diagnosis and initiation of medical management for RA are imperative to reduce the risk of irreversible damage to multiple organ systems in these patients. In the absence of typical clinical manifestations of RA, our case serves as a reminder to maintain a high clinical suspicion of RA in the presence of more unusual cardiac findings - including hemorrhagic pericardial effusions. This case specifically demonstrates the importance of considering hemorrhagic pericardial effusion as a marker of undiagnosed RA, particularly if malignancy or traumatic causes have been ruled out.
